# Combination of Xpert MTB/RIF and TBAg/PHA Ratio for Prompt Diagnosis of Active Tuberculosis: A Two-Center Prospective Cohort Study

**DOI:** 10.3389/fmed.2020.00119

**Published:** 2020-04-15

**Authors:** Feng Wang, Kui Liu, Jing Peng, Ying Luo, Guoxing Tang, Qun Lin, Hongyan Hou, Weiyong Liu, Jing Wang, Zemin Fang, Haobin Kuang, Ziyong Sun

**Affiliations:** ^1^Department of Laboratory Medicine, Tongji Hospital, Tongji Medical College, Huazhong University of Science and Technology, Wuhan, China; ^2^Department of Respiratory and Critical Care Medicine, Tongji Hospital, Tongji Medical College, Huazhong University of Science and Technology, Wuhan, China; ^3^Department of Prevention and Health Care, Jianghan University, Wuhan, China; ^4^Department of Cardiothoracic and Vascular Surgery, Tongji Hospital, Tongji Medical College, Huazhong University of Science and Technology, Wuhan, China; ^5^Department of Tuberculosis, Guangzhou Chest Hospital, Guangzhou, China

**Keywords:** Xpert MTB/RIF, TBAg/PHA ratio, T-SPOT, active tuberculosis, non-tuberculosis

## Abstract

The prompt diagnosis of active tuberculosis (ATB) is still a challenge in clinical practice, especially in TB-endemic countries. We prospectively enrolled consecutive patients with suspected pulmonary TB from two tertiary hospitals. Acid-fast staining (AFS), Xpert MTB/RIF (Xpert), *Mycobacterium tuberculosis* culture, and T-SPOT.TB were simultaneously performed. 226 ATB and 348 non-TB patients were diagnosed in Tongji hospital (test cohort), and 86 ATB and 110 non-TB patients were diagnosed in Guangzhou Chest Hospital (validation cohort). Using ATB as patient group and non-TB as control group, for diagnosis of ATB in Tongji Hospital, the sensitivity of AFS was 17.70% (95% CI: 13.08–23.44%). The sensitivity of Xpert and culture were 53.54% (95% CI: 46.81–60.14%) and 46.46% (95% CI: 39.86–53.19%), respectively. The sensitivity of T-SPOT.TB was 81.42% (95% CI: 75.60–86.14%), but the specificity was 71.55% (95% CI: 66.60–76.04%). Calculation of the ratio of TB-specific antigen to phytohaemagglutinin (TBAg/PHA) of T-SPOT.TB assay increased the specificity but with a loss of sensitivity. Combination of Xpert and culture slightly increased the sensitivity compared to using these methods separately. Combination of Xpert and TBAg/PHA ratio (defined as Xpert positive or TBAg/PHA ≥ 0.2) increased diagnostic accuracy, and the sensitivity and specificity of combination of them were 85.84% (95% CI: 80.45–89.98%) and 95.98% (95% CI: 93.36–97.59%), respectively. The diagnostic model was also established based on combination of Xpert and TBAg/PHA ratio. The area under the curve of the diagnostic model was 0.952 (95% CI: 0.932–0.973) for diagnosis of ATB, with a sensitivity of 88.05% (95% CI: 83.10–91.98%) and a specificity of 96.26% (95% CI: 93.70–98.00%) when a cutoff value of 0.44 was used in Wuhan cohort. The performance of combination of Xpert and TBAg/PHA ratio was similar in Guangzhou Chest Hospital. Our data suggest that combination of Xpert and TBAg/PHA ratio may be a good algorithm for prompt diagnosis of ATB in high endemic areas.

## Introduction

Accurate and prompt diagnosis of tuberculosis (TB) is crucial for patient management and TB control. However, none of the currently used methods can satisfy this requirement. The sensitivity of acid-fast staining (AFS) is low (1, 2). *Mycobacterium tuberculosis* (MTB) culture and Xpert MTB/RIF (Xpert) assay face the same dilemma under low bacterial loads and MTB culture is also limited by a long turnaround time (3–5). Tuberculin skin test and T-SPOT.TB (T-SPOT) have been proven useful in detecting MTB infection (6–9). The great limitation of these methods is their inability to distinguish active TB (ATB) from latent TB infection (LTBI) (8, 10–12).

Our previous studies have found that calculation of the ratio of TB-specific antigen (TBAg) to phytohaemagglutinin (PHA) (TBAg/PHA ratio) of T-SPOT increases the specificity of ATB diagnosis (13–18). The theoretical basis of this method is that TBAg/PHA ratio can eliminate the impact of individual immune variation on T-SPOT. For instance, ATB patients with immunocompromised conditions have decreased TBAg results, leading to increased difficulty in distinguishing ATB from LTBI because low TBAg results are mostly attributed to LTBI. However, PHA results, the positive control of T-SPOT assay, are correspondingly decreased in these situations because they can reflect the immune status of the host (14, 15). Thus, the TBAg/PHA ratio is still in a high level and better than directly using TBAg results in this condition.

The decreased bacillary burden was found in patients with immunosuppression and Xpert cycle threshold values were significantly increased among HIV-infected individuals (19). Thus, Xpert shows reduced sensitivity and negative predictive value in patients with immunosuppression (20). Our previous study has shown that the TBAg/PHA ratio can eliminate immune variation and has some diagnostic value in ATB patients with immunocompromised condition (16). Thus, we speculated that combination of Xpert and TBAg/PHA ratio can improve the diagnostic accuracy of ATB. Both Xpert and TBAg/PHA ratio can be completed within 24 h. In this study, we first determined whether combination of Xpert and TBAg/PHA ratio can be a good algorithm for prompt diagnosis of ATB.

## Materials and Methods

### Study Subjects and Clinical Procedures

According to symptoms (such as cough, fever, or night sweat) and typical computed tomography (CT) findings, the suspected pulmonary TB patients were consecutively recruited from Tongji Hospital (one of the biggest tertiary hospitals in central region of China, located in Wuhan which has 11 million people) and Guangzhou Chest Hospital (a TB-specialized hospital located in Guangzhou which has 14 million people) between October 2017 and October 2018. Patients younger than 18 years of age and those undergoing TB treatment were excluded.

One to three sputum samples were collected from recruited patients if they had cough and all recruited patients underwent fiberoptic bronchoscopy. Bronchial washing fluid (BWF) was collected from the lung segment that showed abnormal lesions on CT. BWF was obtained by instillation of 50–60 ml of saline and at least 30 ml returned aspirate was collected. Sputum and BWF were used for AFS (Ziehl-Neelsen staining) and classified as negative, scanty and 1+, 2+ or 3+ depending on the number of bacilli under the microscope (21). Only BWF was used for performing Xpert MTB/RIF (Cepheid, Sunnyvale, USA) and MTB culture (MGIT 960 and Lowenstein-Jensen media) for cost-effectiveness consideration. MTB isolates were identified by the MPT 64 antigen detection and the line probe assay GenoType MTBDRplus. Blood samples were collected for T-SPOT assay.

Tissue samples were collected from some patients who underwent bronchoscopy if needed. Endobronchial ultrasound-guided transbronchial needle aspiration (EBUS-TBNA) and CT-guided percutaneous lung biopsy (PTLB) were performed in some patients. All tissue samples were examined histologically. If extrapulmonary space-occupying lesions such as shoulder, neck, and pancreatic mass or extrapulmonary lymph node enlargement were found in radiographic examinations, fine-needle aspiration biopsy was performed. Pleural fluid was collected for cytological and biochemical analyses in patients who had pleural effusion. Clinical information and conventional test results were collected from electronic patient records.

All patients with positive MTB results were given anti-TB treatment. If histopathology and cytology support probable ATB, patients were given anti-TB treatment. If no evidence of TB was present, patients were treated with broad-spectrum antibiotics first. If no response was found, patients who had no other diagnoses and were still suspected of having TB were also given anti-TB treatment. All patients received anti-TB treatment should be followed up for 6 months. The clinicians were blind to T-SPOT results when making the above decisions. This study was approved by the ethical committee of Tongji Hospital, Tongji Medical College, Huazhong University of Science and Technology; the ethical committee of Guangzhou Chest Hospital, China.

### Diagnostic Criteria

Cases of ATB were classified as confirmed or probable ATB. Confirmed ATB was defined as having positive results of MTB culture and/or Xpert in BWF. Probable ATB was defined as histological or cytological findings (including pulmonary and extrapulmonary) suggestive of ATB and with response to anti-TB treatment within 6 months. Patients without evidence of ATB but with response to anti-TB treatment following failure to respond to broad-spectrum antibiotics were also classified as probable ATB. Patients were diagnosed as non-TB if other diagnoses were made. Patients did not satisfy the above criteria were classified as without final diagnosis and were excluded for further analysis.

### T-SPOT Assay and TBAg/PHA Ratio

Peripheral blood was collected from all suspected patients. T-SPOT assay (Oxford Immunotec, Oxford, England) was performed according to the manufacturer's instructions. Briefly, the isolated peripheral blood mononuclear cells (PBMCs) (2.5 × 10^5^) were added to 96-well plates precoated with anti-IFN-γ antibody. Four wells were used for each patient: medium well (negative control), PHA well (positive control), TBAg well including early secreted antigenic target 6 (ESAT-6) and culture filtrate protein 10 (CFP-10). Plates were incubated for 16–20 h at 37°C with 5% CO_2_, washed with PBS and developed using an anti-IFN-γ antibody conjugate and substrate to detect the presence of secreted IFN-γ. Spot-forming cells (sfc) were counted with an automated ELISPOT reader (CTL Analyzers, Cleveland, USA). Positive and negative results were defined according to the manufacturer's recommendations. Results were considered undetermined if the spot amounts in the positive control were <20. The ratios of (1) ESAT-6 sfc to PHA sfc, and (2) CFP-10 sfc to PHA sfc were calculated. The larger of the above two values was defined as the TBAg/PHA ratio of one patient.

### Statistical Analysis

The results are presented as mean ± standard deviation (SD). Differences for continuous variables between two groups were analyzed by using the Mann-Whitney *U*-test. The sensitivity and specificity of AFS, Xpert and culture were calculated, and 95% confidence interval (CI) for sensitivity and specificity were determined by using the Wilson score method. Receiver operating characteristic (ROC) analysis was performed for ESAT-6 sfc, CFP-10 sfc, PHA sfc and TBAg/PHA ratio and area under the curve (AUC) was identified, and the sensitivity and specificity were calculated at different cutoff points. AUCs of different indicators were compared by using DeLong test. The combination of Xpert and TBAg/PHA ratio was defined as Xpert positive or TBAg/PHA ≥ cutoff value. A diagnostic model based on combination of Xpert and TBAg/PHA ratio was also established using multivariate logistic regression for ATB diagnosis. Data were analyzed by using SPSS version 19.0 (SPSS, Chicago, IL), GraphPad Prism 6.0 (San Diego, CA, USA), and MedCalc version 11.6 (Medcalc, Mariakerke, Belgium). Statistical significance was determined as *p* **<** 0.05.

## Results

### Patients' Characteristics

**Figure 1:**
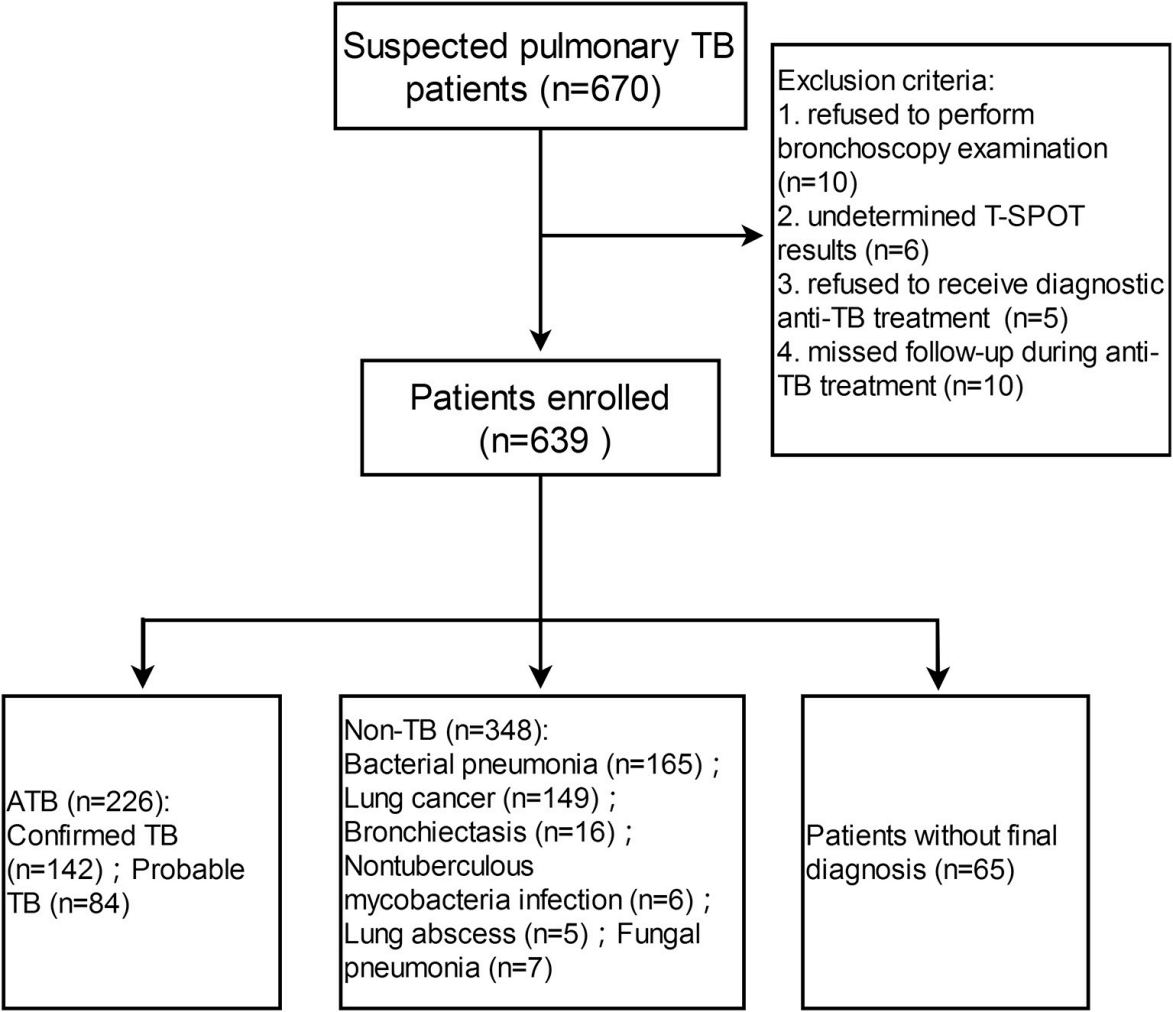
Flow diagram summarizing participant recruitment in test cohort. TB, tuberculosis; ATB, active tuberculosis; T-SPOT, T-SPOT.TB.

A total of 670 patients with suspected pulmonary TB were recruited from Tongji Hospital (test cohort), and 226 and 348 patients (bacterial pneumonia, *n* = 165; lung cancer, *n* = 149; bronchiectasis, *n* = 16;nontuberculous mycobacteria infection, *n* = 6;lung abscess, *n* = 5;fungal pneumonia, *n* = 7) were diagnosed as ATB and non-TB, respectively (Figure 1). Demographic and clinical characteristics of the participants are shown in Table 1. HIV infection was low (3 HIV-positive in 226 ATB and 2 HIV-positive in 348 non-TB) among study participants. Tuberculin skin test was not performed because all participants had a history of bacille Calmette-Guérin vaccination.

**Table 1:** Demographic and clinical characteristics of study participants in Tongji Hospital by classification groups.

**Characteristic**	**ATB (*****n*** **=** **226)**		**Non-TB (*n* = 348)**	**Without final diagnosis (*n* = 65)**
	**Confirmed ATB (*n* = 142)**	**Probable ATB (*n* = 84)**		
Mean age (mean ± SD), years	47.16 ± 16.69	48.02 ± 15.23	50.53 ± 14.30	48.35 ± 16.85
Male sex	82 (57.75)	49 (58.33)	191 (54.89)	36 (55.38)
History of TB	20 (14.08)	13 (15.48)	10 (2.87)	1 (1.54)
**Symptoms at enrollment**				
Cough	125 (88.03)	72 (85.71)	286 (82.18)	60 (92.31)
Fever	85 (59.86)	40 (47.62)	175 (50.29)	31 (47.69)
Breathing difficulties	56 (39.44)	31 (36.90)	101 (29.02)	21 (32.31)
Fatigue	48 (33.80)	26 (30.95)	105 (30.17)	11 (16.92)
Chest pain	41 (28.87)	23 (27.38)	89 (25.57)	10 (15.38)
Haemoptysis	18 (12.68)	8 (9.52)	45 (12.93)	1 (1.54)
Night sweats	21 (14.79)	8 (9.52)	29 (8.33)	4 (6.15)
Weight loss	28 (19.72)	12 (14.29)	61 (17.53)	8 (12.31)
Abdominal pain or shoulder pain	10 (7.04)	3 (3.57)	13 (3.74)	1(1.54)
**Pulmonary radiological findings**				
Shadow	124 (87.32)	72 (85.71)	285 (81.90)	55 (84.62)
Nodules or masses	90 (63.38)	56 (66.67)	188 (54.02)	41 (63.08)
Lymph node enlargement or hyperplasia	73 (51.41)	38 (45.24)	199 (57.18)	35 (53.85)
Cavitation	38 (26.76)	17 (20.24)	38 (10.92)	11 (16.92)
Pleural thickening or adhesion	42 (29.58)	26 (30.95)	88 (25.29)	18 (27.69)
Emphysema	15 (10.56)	10 (11.90)	68 (19.54)	6 (9.23)
Bronchiectasis	3 (2.11)	5 (5.95)	36 (10.34)	3 (4.62)
Calcification	19 (13.38)	10 (11.90)	55 (15.80)	9 (13.85)
Pleural effusion	21 (14.79)	20 (23.81)	45 (12.93)	1 (1.54)
**Underlying conditions**				
Hypertension	12 (8.45)	10 (11.90)	31 (8.91)	7 (10.77)
HIV infection	0	3 (3.57)	2 (0.57)	0
Hematological malignancy receiving chemotherapy	2 (1.41)	2 (2.38)	3 (0.86)	0
Chronic renal failure	4 (2.82)	3 (3.57)	8 (2.30)	1 (1.54)
Liver cirrhosis	1 (0.70)	1 (1.19)	2 (0.57)	0
Autoimmune disease receiving treatment	4 (2.82)	4 (4.76)	4 (1.15)	1 (1.54)
Transplantation receiving treatment	2 (1.41)	3 (3.57)	2 (0.57)	0
Diabetes	5 (3.52)	7 (8.33)	9 (2.59)	3 (4.62)

*Data are presented as numbers (%) unless otherwise indicated. TB, tuberculosis; ATB, active tuberculosis; SD, standard deviation; AFS, acid-fast staining; MTB, mycobacterium tuberculosis; T-SPOT, T-SPOT.TB*.

**Figure 2:**
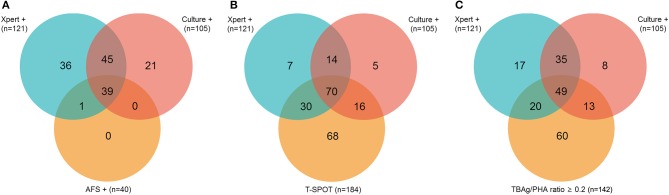
Venn diagrams showing the overlap of positive Xpert, culture, and AFS **(A)** or Xpert, culture, and T-SPOT **(B)** or Xpert, culture, and TBAg/PHA ratio **(C)** in ATB patients. AFS, acid-fast staining; T-SPOT, T-SPOT.TB; TBAg/PHA ratio, the ratio of TB-specific antigen to phytohaemagglutinin.

Of 226 ATB patients, 142 (62.83%) and 84 (37.17%) were diagnosed as confirmed and probable ATB, respectively. In confirmed ATB patients, 40 (28.17%) had positive AFS; 121 (85.21%) had positive Xpert; 105 (73.94%) had positive culture; and 184 (81.42%) had positive T-SPOT. No rifampicin resistance was detected in Xpert. The overlap between ATB patients with a positive Xpert, culture, or AFS is shown in Figure 2A. The overlap between ATB patients with a positive Xpert, culture, or T-SPOT is shown in Figure 2B. All AFS positive samples were Xpert positive.

Of 142 confirmed ATB patients, 88 (61.97%) had samples of sputum and BWF, and 54 (38.03%) only had sample of BWF. In AFS positive patients, BWF positive but sputum negative (47.50%) or BWF alone positive (30%) was more common than sputum positive but BWF negative (5%). In 9 sputum AFS positive patients, the proportion of three sputum samples was higher than one sputum sample (Supplementary Table 1).

Of 84 probable ATB patients, 53 (63.10%) had samples of sputum and BWF, and 31 (36.90%) only had sample of BWF. In these patients, 54 (64.29%) had pathological results consistent with ATB (bronchoscopy biopsy, 20; EBUS-TBNA lymph node biopsy, 12; CT-guided PTLB biopsy, 13; pleural tissue biopsy, 2; surgical resection biopsy, 1; extra-pulmonary tissue or lymph node biopsy, 6), 14 (16.67%) had pleural fluid cytological and biochemical indexes (lymphocyte% ≥ 75%; adenosine deaminase ≥ 30 IU/L; lactate dehydrogenase ≥ 200 U/L) consistent with ATB. All probable ATB patients had successful response to 6 months of anti-TB treatment.

### TBAg/PHA Ratio

**Figure 3:**
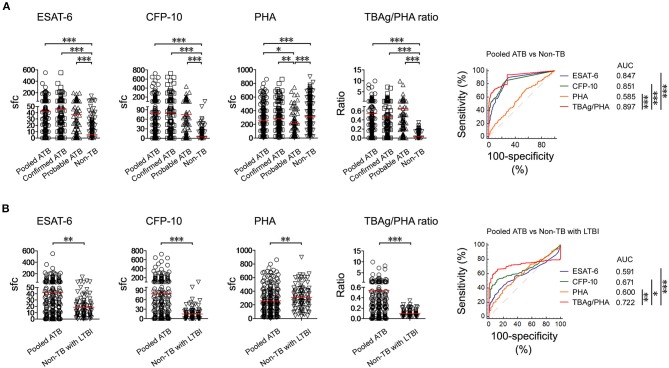
T-SPOT results in distinguishing ATB from non-TB. **(A)** Dot plots showing ESAT-6 sfc, CFP-10 sfc, PHA sfc, and TBAg/PHA ratio in pooled ATB (*n* = 226), confirmed ATB (*n* = 142), probable ATB (*n* = 84), and non-TB (*n* = 348). ROC curves showing the performance of these markers in distinguishing pooled ATB from non-TB. **(B)** Dot plots showing ESAT-6 sfc, CFP-10 sfc, PHA sfc, and TBAg/PHA ratio in pooled ATB (*n* = 226) and non-TB with LTBI (*n* = 99). ROC curves showing the performance of these markers in distinguishing pooled ATB from non-TB with LTBI. Bars indicate means. Each symbol represents an individual donor. *p < 0.05, **p < 0.01, ***p < 0.001 (Mann–Whitney *U*-test). TB, tuberculosis; ATB, active tuberculosis; LTBI, latent tuberculosis infection; ESAT-6, early secreted antigenic target 6; CFP-10, culture filtrate protein 10; PHA, phytohaemagglutinin; TBAg/PHA ratio, the ratio of TB-specific antigen to phytohaemagglutinin; sfc, spot-forming cells; AUC, area under the curve.

Both ESAT-6 (42.29 ± 64.24) and CFP-10 (79.22 ± 119.7) sfc in pooled ATB patients were significantly higher than those in non-TB patients (ESAT-6, 5.31 ± 13.39; CFP-10, 4.33 ± 12.17). In contrast, PHA (258.4 ± 187.0) sfc was significantly decreased in pooled ATB patients compared with non-TB patients (310.7 ± 190.9). The TBAg/PHA ratio (0.541 ± 1.156) in pooled ATB patients was remarkably higher than in non-TB patients (0.027 ± 0.055). ROC analysis showed that TBAg/PHA ratio performed better than ESAT-6 or CFP-10 in distinguishing pooled ATB from non-TB (Figure 3A). The AUC of the ROC curve for TBAg/PHA ratio was 0.897 (95% CI, 0.870–0.921).

The aim of the study was to further differentiate between ATB and non-TB with LTBI (patients who had other diagnoses and with positive T-SPOT). Similarly, the TBAg/PHA ratio in ATB patients was significantly higher than that in non-TB patients with LTBI (0.093 ± 0.064). The performance of TBAg/PHA ratio was decreased in this situation, but was still highest among these parameters. The AUC of the ROC curve for TBAg/PHA ratio was 0.722 (95%, 0.670–0.770) (Figure 3B). Using the cutoff value of 0.207, the sensitivity and specificity of TBAg/PHA ratio in distinguishing these two conditions were 61.50% and 90.91%, respectively.

### Classification Groups

We selected 0.2 as the cutoff value of the TBAg/PHA ratio. Figure 2C shows the overlap between pooled ATB patients with positive Xpert, culture, or TBAg/PHA ratio≥0.2. Table 2 shows the results of different methods in classification groups. As a single test for diagnosis of confirmed ATB, the sensitivity of Xpert was highest (85.21%). The sensitivity of T-SPOT (81.69%) was higher than TBAg/PHA ratio (57.75%), while the specificity of TBAg/PHA ratio (95.98%) was higher than T-SPOT (71.55%). Furthermore, combination of Xpert and TBAg/PHA ratio (defined as Xpert positive or TBAg/PHA≥0.2) had both high sensitivity (94.37%) and high specificity (95.98%) for diagnosis of confirmed ATB (Table 2).

**Table 2:** The results of different methods in classification groups.

	**Confirmed ATB (*n* = 142)**	**Probable ATB (*n* = 84)**	**Non-TB (*n* = 348)**	**Without final diagnosis (*n* = 65)**
AFS+	40 (28.17%)	0	6 (1.72%)	0
Xpert+	121 (85.21%)	0	0	0
Culture+	105 (73.94%%)	0	0	0
T-SPOT+	116 (81.69%)	68 (80.95%)	99 (28.45%)	16 (24.61%)
TBAg/PHA ≥ 0.2	82 (57.75%)	60 (71.43%)	14 (4.02%)	5 (7.69%)
Xpert+ or TBAg/PHA ≥ 0.2	134 (94.37%)	60 (71.43%)	14 (4.02%)	5 (7.69%)

*T-SPOT: T-SPOT.TB; TB: tuberculosis; ATB: active tuberculosis; TBAg/PHA: the ratio of TB-specific antigen to phytohaemagglutinin; +: positive*.

### Using Different Methods for the Diagnosis of Pooled ATB

As a single test for diagnosis of pooled ATB, the sensitivity and specificity of AFS, Xpert, MTB culture, T-SPOT, and TBAg/PHA ratio were 17.70 and 98.28%, 53.54 and 100%, 46.46 and 100%, 81.42 and 71.55%, and 62.83 and 95.98%, respectively. Combination of Xpert and AFS had no improvement compared with Xpert alone. Combination of Xpert and culture slightly increased the diagnostic sensitivity to 62.83%. Combination of Xpert and T-SPOT showed high sensitivity (90.71%) but limited specificity (71.55%). Combination of Xpert and TBAg/PHA ratio showed both high sensitivity (85.84%) as well as high specificity (95.98%) (Table 3).

**Table 3:** The performance of different methods in the diagnosis of active pulmonary TB in Tongji Hospital.

	**Sensitivity % (95% CI)**	**Positive/total**	**Specificity % (95% CI)**	**Negative/total**	**Positive predictive value (%)**	**Negative predictive value (%)**
AFS+	17.70 (13.08–23.44)	40/226	98.28 (96.29–99.21)	342/348	87	64.77
Xpert+	53.54 (46.81–60.14)	121/226	100 (98.91–100)	348/348	100	76.82
Culture+	46.46 (39.86–53.19)	105/226	100 (98.91–100)	348/348	100	74.20
T-SPOT+	81.42 (75.60–86.14)	184/226	71.55 (66.60–76.04)	249/348	65.02	85.57
TBAg/PHA ≥ 0.2	62.83 (56.14–69.08)	142/226	95.98 (93.36–97.59)	334/348	91.03	79.90
Xpert+ or AFS+	53.54 (46.81–60.14)	121/226	98.28 (96.29–99.21)	342/348	95.28	76.51
Xpert+ or culture+	62.83 (56.14–69.08)	142/226	100 (98.91–100)	348/348	100	80.56
Xpert+ or T-SPOT+	90.71 (85.96–94.02)	205/226	71.55 (66.60–76.04)	249/348	67.43	92.22
Xpert+ or TBAg/PHA ≥ 0.2	85.84 (80.45–89.98)	194/226	95.98 (93.36–97.59)	334/348	93.27	91.26
Diagnostic model*	88.05 (83.1–91.98)	199/226	96.26% (93.7–98)	335/348	93.87	92.54

* *Diagnostic model was established by combination of Xpert and TBAg/PHA ratio. TB, tuberculosis; AFS, acid-fast staining; Xpert, Xpert MTB/RIF; T-SPOT, T-SPOT.TB; TBAg/PHA ratio, the ratio of TB-specific antigen to phytohaemagglutinin; CI, confidence interval; +, positive*.

**Figure 4:**
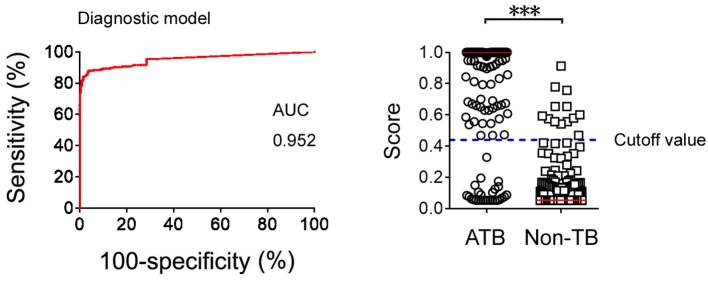
The results of diagnostic model based on combination of Xpert and TBAg/PHA ratio. ROC analysis showing the performance of the diagnostic model based on combination of Xpert and TBAg/PHA ratio in distinguishing ATB from non-TB. Scatter plots showing the score of diagnostic model based on combination of Xpert and TBAg/PHA ratio in ATB (*n* = 226) and non-TB (*n* = 348) patients. Horizontal lines indicate the median. ***P < 0.001 (Mann-Whitney *U*-test). Blue dotted line indicates the cutoff value in distinguishing these two groups. TB, tuberculosis; ATB, active tuberculosis; AUC, area under the curve.

Logistic regression analysis was also used to determine whether combination of Xpert and TBAg/PHA ratio can improve the diagnostic effect. The diagnostic model was obtained as the following: P = 1/[1 + e^−(−2.892 + 15.204 × *TBAg*/*PHA* + 22.205 × *Xpert*)^] P, predictive value; e, natural logarithm. Xpert was given a score of 1 if positive and 0 if negative. ROC analysis showed that the AUC of the diagnostic model was 0.952 (95% CI: 0.932–0.973) for diagnosis of ATB, with a sensitivity of 88.05% and a specificity of 96.26% when a cutoff value of 0.44 was used (Figure 4).

### Validation Cohort

A total of 229 patients with suspected pulmonary TB were recruited from Guangzhou Chest Hospital (validation cohort). Flow diagram summarizes the patient recruitment (Supplementary Figure 1). Demographic and clinical characteristics of the validation cohort are shown in Supplementary Table 2. The performance of combination of Xpert and TBAg/PHA ratio in validation cohort was comparable to that in test cohort (Table 4).

**Table 4:** The performance of different methods in the diagnosis of active pulmonary TB in Guangzhou Chest Hospital.

	**Sensitivity % (95% CI)**	**Positive/total**	**Specificity % (95% CI)**	**Negative/total**	**Positive predictive value (%)**	**Negative predictive value (%)**
AFS+	19.77 (12.72–29.40)	17/86	100 (96.63–100)	110/110	100	61.45
Xpert+	56.98 (46.44–66.92)	49/86	100 (96.63–100)	110/110	100	74.83
Culture+	55.84 (45.29–65.84)	48/86	100 (96.63–100)	110/110	100	74.32
T-SPOT+	84.88 (75.84–90.95)	73/86	71.82 (62.79–79.38)	79/110	70.19	85.87
TBAg/PHA≥0.2	61.63 (51.06–71.20)	53/86	92.73 (86.30–96.27)	102/110	86.89	75.56
Xpert+ or AFS+	56.98 (46.44–66.92)	49/86	100 (96.63–100)	110/110	100	74.83
Xpert+ or culture+	62.79 (52.23–72.25)	54/86	100 (96.63–100)	110/110	100	77.46
Xpert+ or T-SPOT+	90.70 (82.70–95.21)	78/86	71.82 (62.79–79.38)	79/110	71.56	90.80
Xpert+ or TBAg/PHA≥0.2	86.05 (77.18–91.83)	74/86	92.73 (86.30–96.27)	102/110	90.24	89.47
Diagnostic model*	88.37 (79.9–93.56)	76/86	93.64 (87.44–96.88)	105/110	91.57	91.15

* *Diagnostic model was established by combination of Xpert and TBAg/PHA ratio. TB, tuberculosis; AFS, acid-fast staining; Xpert, Xpert MTB/RIF; T-SPOT, T-SPOT.TB; TBAg/PHA, the ratio of TB-specific antigen to phytohaemagglutinin; CI, confidence interval; +, positive*.

## Discussion

Accurate and prompt diagnosis of active pulmonary TB is still a challenge in clinical practice. This is especially difficult in TB-endemic countries since LTBI can cause interference. In this study, we hypothesized that combination of Xpert and TBAg/PHA ratio may be an effective way to solve this dilemma.

One of the characteristics of this study is that each enrolled patient underwent bronchoscopy. Flexible bronchoscopy is a useful tool in diagnosing pulmonary TB, as it can be utilized to obtain respiratory samples in patients with sputum AFS negative or who cannot expectorate sputum (22–24). In addition, BWF is easier to obtain than bronchial alveolar lavage fluid (25). Thus, at least one sample of BWF was collected from each enrolled patient. As expected, our results showed that BWF is obviously better than sputum in detecting MTB (Supplementary Table 1). In accordance with previous studies, it is common to see Xpert positive but culture negative, or culture positive but Xpert negative results in clinically even using the same sample (20, 26, 27). Unexpectedly, we observed that the sensitivity of culture seemed to be lower than that of Xpert in test cohort. The digestion of BWF may be one of the reasons for decreased sensitivity of culture. Furthermore, lidocaine used at bronchoscopy for topical anesthesia can inhibit MTB growth in culture, which may also decrease the sensitivity of culture when using BWF as the sample source (28).

The currently available TB detection methods all have some limitations (29–34). Our studies have shown that calculation of TBAg/PHA ratio in T-SPOT improves the specificity, but actually sacrifices the sensitivity. Theoretically, Xpert focuses on the detection of MTB pathogen, but TBAg/PHA ratio detects MTB-specific lymphocyte responses of hosts. These two methods can make up for each other's shortcomings based on detecting different aspects. Second, the sensitivity of Xpert is decreased in patients with immunosuppression because of low MTB burden, while PHA value in T-SPOT is decreased and TBAg/PHA ratio still maintains a high level in this condition. Third, although the sensitivity of Xpert could reach up to 80% (84/105) in culture positive patients, there was a small group of patients with Xpert negative but culture positive results. The TBAg/PHA ratio can play a complementary role in this condition. Thus, combination of Xpert and TBAg/PHA ratio can improve the diagnostic accuracy of the tests.

The diagnosis of probable ATB is very difficult in some cases, as there is no diagnostic standard until now. The proportion of probable ATB in total ATB is relatively low in our two cohort populations (both less than 40%). We have tried our best to find evidence of TB in suspected patients, and most of probable ATB are diagnosed not only based on histopathological or cytological results, but also on successful response to anti-TB treatment.

An interesting question is why TBAg/PHA ratio in probable ATB is higher than in confirmed ATB. As discussed above, we speculate that TBAg/PHA ratio is negatively correlated with MTB burden because PHA results are deceased in patients with immunosuppression. This could be the most important reason that TBAg/PHA ratio is suitable for detection of probable ATB. Two facts can support this hypothesis. First, in the present study, the TBAg/PHA ratio was gradually declined in patients (*n* = 40) with scanty, 1+, 2+, and 3+ AFS results (Supplementary Figure 2A). We also retrospectively analyzed AFS results in our laboratory in past 5 years. Our data showed that the TBAg/PHA ratio was also negatively correlated with AFS semi-quantitative results (*n* = 480) (Supplementary Figure 2B). However, we did not observe a similar trend in either ESAT-6 or CFP-10 results (Supplementary Figures 2C,D). Thus, the TBAg/PHA ratio is negatively associated with MTB burden, which results in a higher TBAg/PHA ratio in probable ATB. Second, the percentage of underlying conditions in probable ATB patients was higher than in ATB patients (Table 1), which might be used to explain why MTB burden in probable ATB is low.

How to count PHA accurately is the key issue of this study, as PHA results are used to calculate TBAg/PHA ratio. The following two procedures are the key factors affected PHA results as discussed in our previous study (15). First, the substrate incubation time should be strictly limited to 7 min (room temperature). If the substrate incubation time is prolonged, PHA results are obviously increased (Supplementary Figure 3A). Second, peripheral blood mononuclear cells should be counted and diluted accurately. With more cells added to plate, PHA sfc is obviously increased (Supplementary Figure 3B).

Another important factor we did not discuss before is the setting of ELISPOT reader parameters. Many parameters such as sensitivity, exposure time, and spot size can affect the results of spot reading. Among these parameters, exposure setting and sensitivity are two key factors. First, auto-exposure model for each well is better than fixed exposure value because auto-model can automatically balance background value. Second, the sensitivity setting of ELISPOT reader should be appropriate. Representative dot plots showed that PHA spot number is accordingly increased with the increase of ELISPOT reader sensitivity (Supplementary Figure 4). We found CTL ELISPOT reader can count spot accurately if spot number is lower than 800 (Supplementary Figure 5). As mean PHA value is 300 in our laboratory, we believe most PHA results are believable. Overall, to achieve global standardization of T-SPOT assay, we still have a long way to go.

Some limitations of the study should be mentioned. First, whether the TBAg/PHA ratio has the same advantage in TB low-burden countries is unknown. Second, the number of HIV-infected patients was very few in this study, and whether combination of Xpert and TBAg/PHA ratio has the same effect in high HIV-prevalence settings is unknown. Third, although we have tried our best to diagnose suspected patients, patients without final diagnosis are still existed, which could lead to bias. Fourth, although combination of Xpert and TBAg/PHA ratio increases the diagnostic accuracy of ATB, the experiment procedures will be relatively complicated. Moreover, the manpower and financial cost will be increased if performing these tests simultaneously, which could also limit the use of combination of these two methods in resource-limited countries.

Taken together, these data indicate that, the sensitivity of AFS is low for diagnosis of ATB. The sensitivity of Xpert and culture are moderate. The sensitivity of T-SPOT is relatively high, but the specificity is limited. Calculation of the TBAg/PHA ratio increases the specificity of T-SPOT but with a loss of sensitivity. Finally, combination of Xpert and TBAg/PHA ratio shows both high sensitivity and specificity, which suggests that combination of these two methods may be a good algorithm for prompt diagnosis of ATB in high endemic areas.

## Data Availability Statement

The raw data supporting the conclusions of this article will be made available by the authors, without undue reservation, to any qualified researcher.

## Ethics Statement

This study was approved by the ethical committee of Tongji Hospital, Tongji Medical College, Huazhong University of Science and Technology; the ethical committee of Guangzhou Chest Hospital, China. The patients/participants provided their written informed consent to participate in this study.

## Author Contributions

FW, ZF, HK, and ZS: conception and design. FW, KL, and JP: data acquisition. YL, GT, QL, HH, JW, and WL: analysis and interpretation. FW and ZS: manuscript preparation.

### Conflict of Interest

The authors declare that the research was conducted in the absence of any commercial or financial relationships that could be construed as a potential conflict of interest.

## Supplementary Material

Supplementary Figure 1 Flow diagram summarizing participant recruitment in validation cohort. TB, tuberculosis; ATB, active tuberculosis; T-SPOT, T-SPOT.TB.

Supplementary Figure 2 Relationship between T-SPOT results and AFS semi-quantitative results. **(A)** The TBAg/PHA ratio in scanty (*n* = 11), 1+ (*n* = 20), 2+ (*n* = 7), and 3+ (*n* = 2) AFS positive patients recruited in this study was shown. **(B)** The TBAg/PHA ratio in scanty (*n* = 179), 1+ (*n* = 172), 2+ (*n* = 93), and 3+ (*n* = 36) AFS positive patients retrospectively collected from electronic patient records in past five years was shown. **(C)** The ESAT-6 and CFP-10 sfc in scanty (*n* = 11), 1+ (*n* = 20), 2+ (*n* = 7), and 3+ (*n* = 2) AFS positive patients recruited in this study was shown. **(D)** The ESAT-6 and CFP-10 sfc in scanty (*n* = 179), 1+ (*n* = 172), 2+ (*n* = 93), and 3+ (*n* = 36) AFS positive patients retrospectively collected from electronic patient records in past five years was shown. Bars indicate means. Each symbol represents an individual donor. ESAT-6, early secreted antigenic target 6; CFP-10, culture filtrate protein 10; TBAg/PHA ratio, the ratio of TB-specific antigen to phytohaemagglutinin; AFS, acid-fast staining; sfc, spot-forming cells.

Supplementary Figure 3 The effects of substrate incubation time and cell count on PHA sfc results. **(A)** Two representative pictures showing substrate incubation time of T-SPOT was set at 7 and 20 min. **(B)** PBMCs were separated from two individuals. Different numbers of cells (2.5 × 10^5^, 5 × 10^5^, 10 × 10^5^, and 20 × 10^5^) were added to T-SPOT plate. Two representative samples showing the PHA sfc under different number of cells per well. PHA, phytohaemagglutinin; sfc, spot-forming cells.

Supplementary Figure 4 The same PHA well of T-SPOT is counted by ELISPOT reader in different sensitivity (140, 150, and 160).

Supplementary Figure 5 **(A)** Representative figures showing that the number of PHA spot is counted by ELISPOT reader between 100 and 800. **(B)** Representative figures showing that ELISPOT reader cannot read PHA spot accurately when the spots are full of T-SPOT well.

Supplementary Table 1 The sample sources and AFS results in 142 confirmed ATB patients of Tongji Hospital.

Supplementary Table 2 Demographic and clinical characteristics of study participants in Guangzhou Chest Hospital by classification groups.
