# Supplementary figures and images for: Combination of Xpert MTB/RIF and TBAg/PHA Ratio for Prompt Diagnosis of Active Tuberculosis: A Two-Center Prospective Cohort Study
Source: Front Med (Lausanne). 2020 Apr 15;7:119. doi: 10.3389/fmed.2020.00119 (PMC7174554; doi:10.3389/fmed.2020.00119)

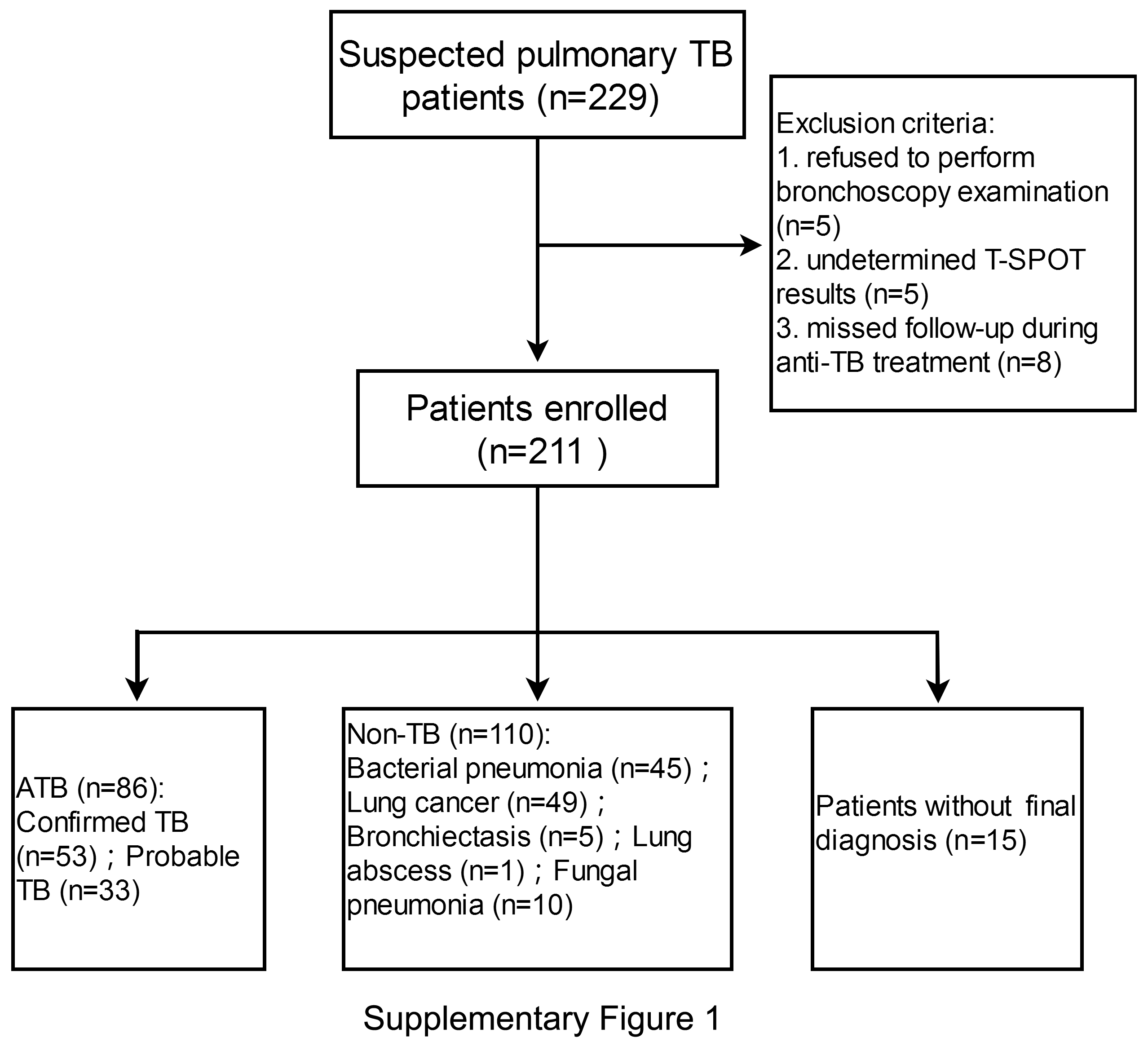

Supplement: Supplementary Figure 1 — Flow diagram summarizing participant recruitment in validation cohort. TB, tuberculosis; ATB, active tuberculosis; T-SPOT, T-SPOT.TB. [file Image_1.TIF]

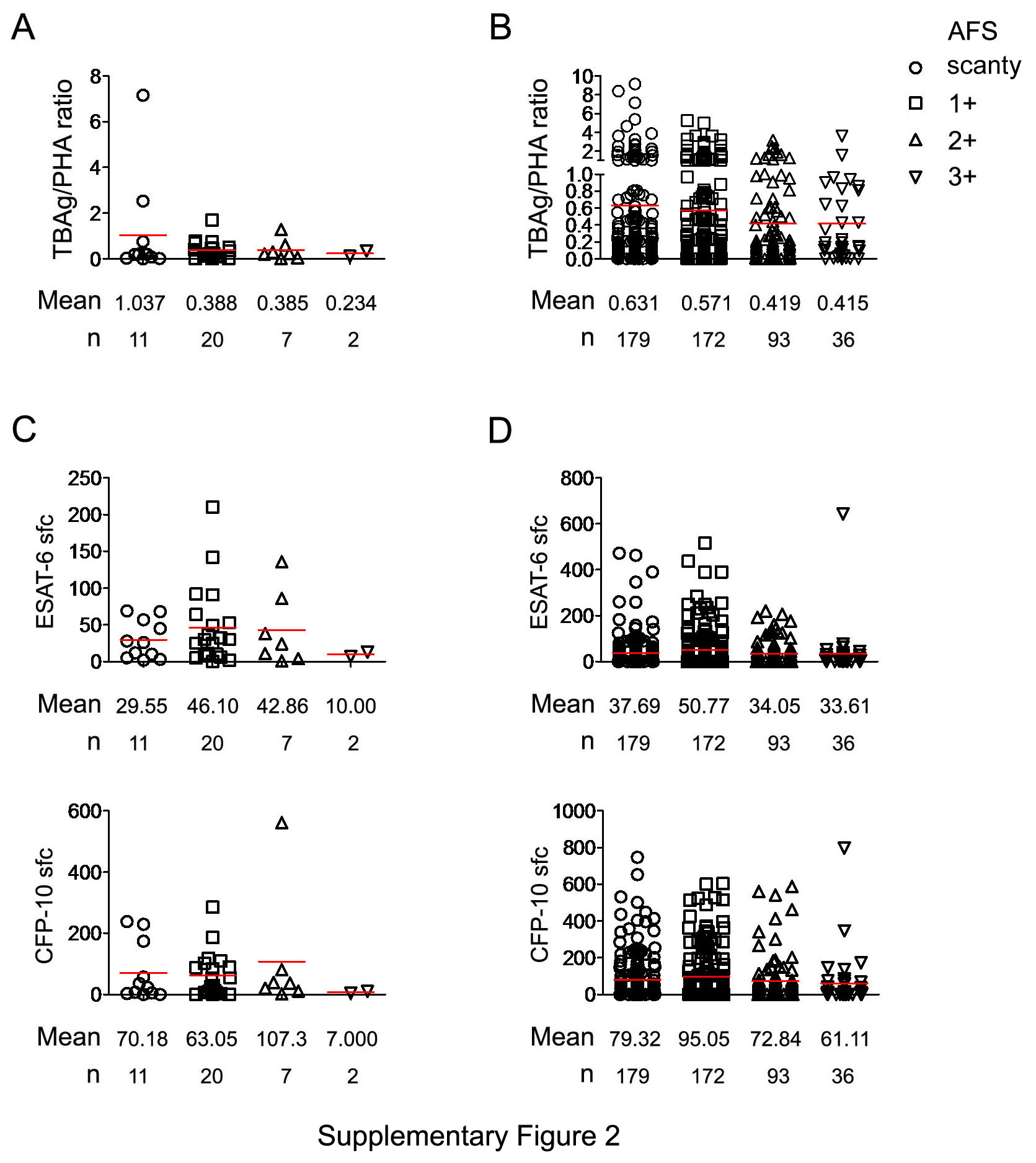

Supplement: Supplementary Figure 2 — Relationship between T-SPOT results and AFS semi-quantitative results. (A) The TBAg/PHA ratio in scanty (n = 11), 1+ (n = 20), 2+ (n = 7), and 3+ (n = 2) AFS positive patients recruited in this study was shown. (B) The TBAg/PHA ratio in scanty (n = 179), 1+ (n = 172), 2+ (n = 93), and 3+ (n = 36) AFS positive patients retrospectively collected from electronic patient records in past five years was shown. (C) The ESAT-6 and CFP-10 sfc in scanty (n = 11), 1+ (n = 20), 2+ (n = 7), and 3+ (n = 2) AFS positive patients recruited in this study was shown. (D) The ESAT-6 and CFP-10 sfc in scanty (n = 179), 1+ (n = 172), 2+ (n = 93), and 3+ (n = 36) AFS positive patients retrospectively collected from electronic patient records in past five years was shown. Bars indicate means. Each symbol represents an individual donor. ESAT-6, early secreted antigenic target 6; CFP-10, culture filtrate protein 10; TBAg/PHA ratio, the ratio of TB-specific antigen to phytohaemagglutinin; AFS, acid-fast staining; sfc, spot-forming cells. [file Image_2.TIF]

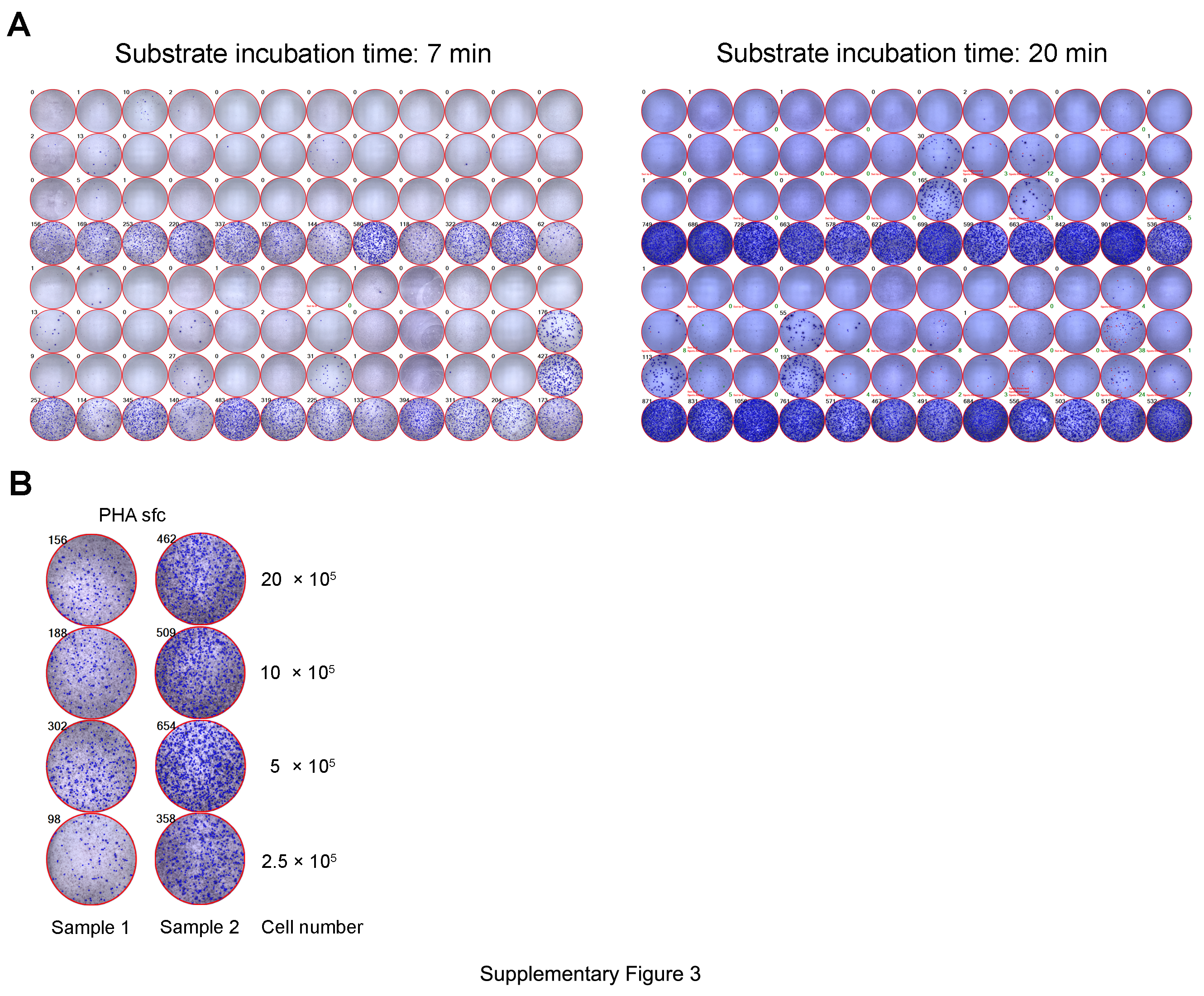

Supplement: Supplementary Figure 3 — The effects of substrate incubation time and cell count on PHA sfc results. (A) Two representative pictures showing substrate incubation time of T-SPOT was set at 7 and 20 min. (B) PBMCs were separated from two individuals. Different numbers of cells (2.5 × 10^5^, 5 × 10^5^, 10 × 105, and 20 × 105) were added to T-SPOT plate. Two representative samples showing the PHA sfc under different number of cells per well. PHA, phytohaemagglutinin; sfc, spot-forming cells. [file Image_3.TIF]

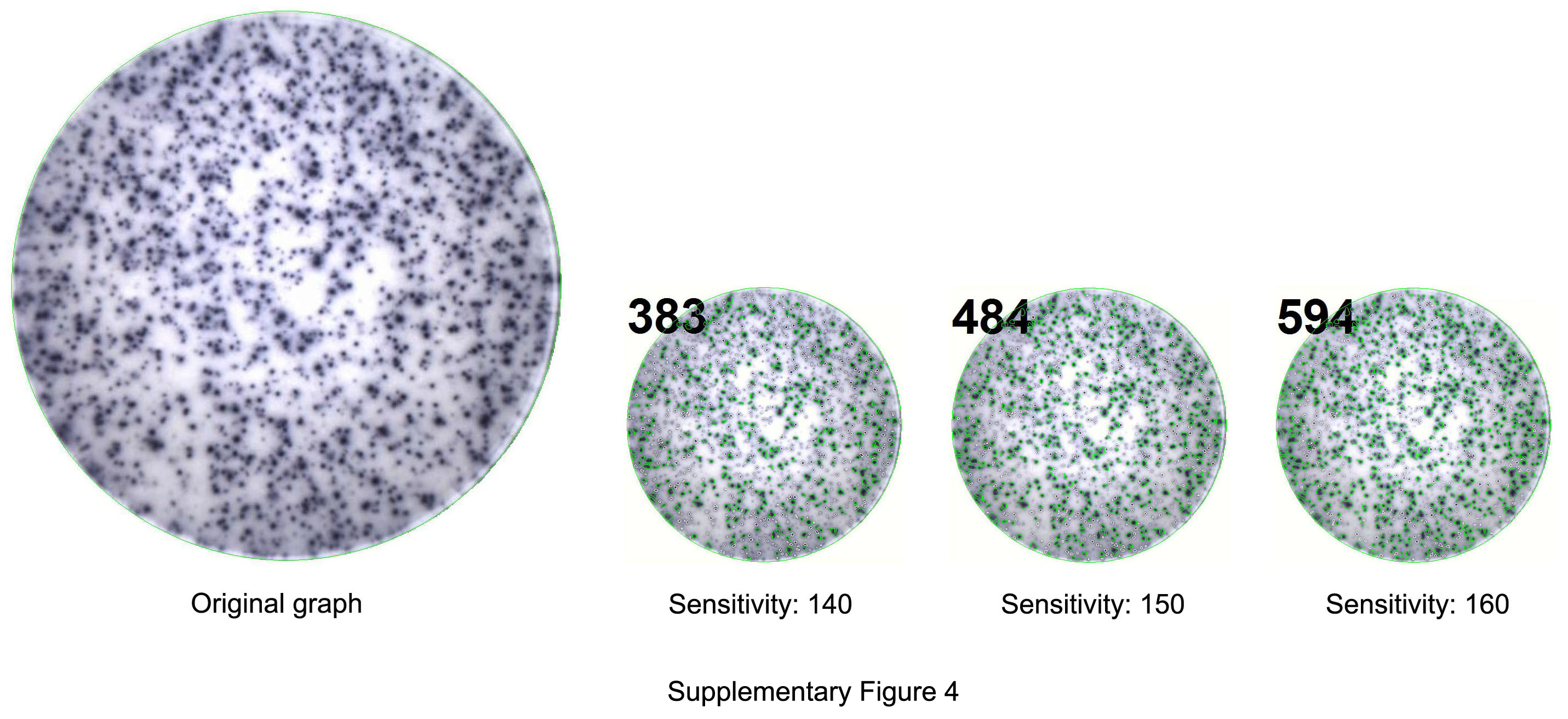

Supplement: Supplementary Figure 4 — The same PHA well of T-SPOT is counted by ELISPOT reader in different sensitivity (140, 150, and 160). [file Image_4.TIF]

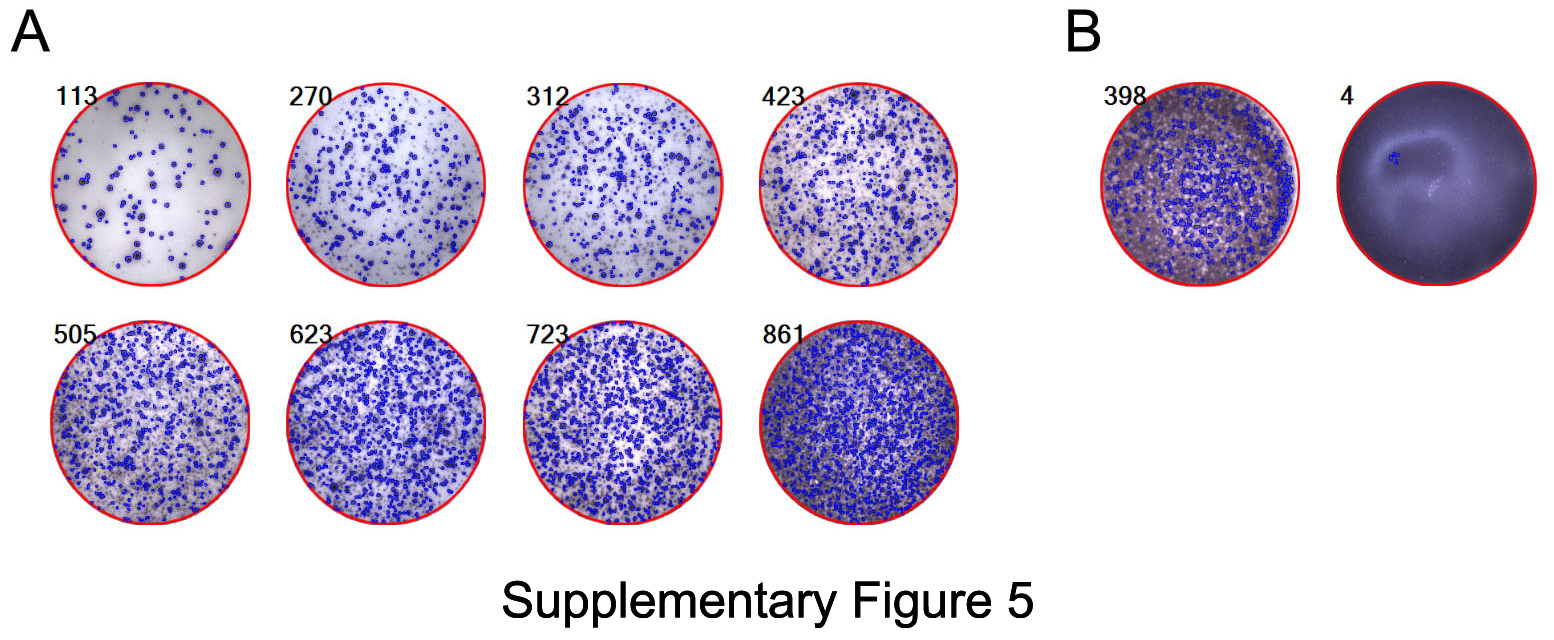

Supplement: Supplementary Figure 5 — (A) Representative figures showing that the number of PHA spot is counted by ELISPOT reader between 100 and 800. (B) Representative figures showing that ELISPOT reader cannot read PHA spot accurately when the spots are full of T-SPOT well. [file Image_5.TIF]
